# Advances in Prostate Cancer Research and Treatment

**DOI:** 10.1155/2014/708383

**Published:** 2014-08-18

**Authors:** Lorenzo Livi, Andrea M. Isidori, David Sherris, Giovanni Luca Gravina

**Affiliations:** ^1^Radiotherapy Unit, Department of Experimental and Clinical Biomedical Sciences, University of Florence, Largo Brambilla 3, 50134 Firenze, Italy; ^2^Department of Experimental Medicine, Sapienza University, 00161 Rome, Italy; ^3^RestorGenex Pharmaceuticals, Inc., 37 Neillian Crescent, Jamaica Plain, MA 02130, USA; ^4^Laboratory of Radiobiology and Division of Radiotherapy, Department of Applied, Clinical and Biotechnological Sciences, University of L'Aquila, Via Vetoio, Coppito 2, 67100 L'Aquila, Italy

It is becoming a truism to state that the progress in computer technologies and nanotechnologies, biomedical imaging, and molecular biology has made it possible to switch from a population treatment approach to a concept based on personalized medicine [1]. The shift from population to individual patient treatment implies the use of information derived from different actors and disciplines which individually do not have the capacity to propose a comprehensive offer [2]. This is particularly true in the field of radiation and medical oncology as well as in clinical and molecular radiology. The main advantage of combining information derived from different clinical and preclinical fields lies in the possibility of selecting a specific population of subjects who, most likely, will benefit from a particular pharmacological or nonpharmacological treatment in accordance with their “molecular profile” at a given time-point [1, 2]. At the same time this information may conversely be used to select patients for whom the risk of adverse effects may be higher [1, 2].

Prostate cancer (Pca) is one of the most commonly diagnosed cancers in men and surgery [3] and radiotherapy (RT) [3–5] remain the gold standard for the treatment of localized or locally advanced Pca. Radiotherapy is configured as a powerful treatment approach with outstanding oncological results and with impressive technical improvements over the last two decades [6]. We now have a greater understanding of mechanisms sustaining the biological processes responsible for tumor progression [7–12] or towards a biological aggressive or radio resistant phenotype [13–15]. However, we are aware that the improvement in oncological outcome of men who remain at high risk for systemic failure may be achieved by improving each diagnostic and therapeutic step including the diagnostic performances of conventional imaging modalities [16]. To date, conventional anatomic imaging techniques of computed tomography (CT), ultrasound, magnetic resonance imaging (MRI), single-photon emission computed tomography (SPECT), and positron emission tomography (PET) are currently used in the common clinical practice to stage men suffering from Pca [17–20]. All these diagnostic tools have peculiar advantages and disadvantages although they play a rather limited role in monitoring men with Pca [17–20]. These limitations are attributable to the incapacity to distinguish malignant from the surrounding nonmalignant tissue [16–20]. The close integration between molecular biology and clinical imaging may ease the development of new molecular imaging agents useful in monitoring a number of biological events that, until a few years ago, were studied by conventional molecular assays [17]. With regard to Pca, progress in quantification, characterization, and timing of biological processes may be obtained overcoming problems related to the amplification of low level signals of in vivo biological events, the development of integrated imaging platforms with sufficiently high spatial and temporal resolution [18], and the need to reach the target in vivo to achieve satisfactory specificity [16–20].

The advances in the molecular based approaches in radiology are specifically evident in oncological treatments [19]. One of the most striking examples of foregoing statements is attested by the development of the enormous amount of specific drugs and inhibitors, the ability to genetically modify cellular systems, and the introduction of a multitude of diagnostic tools able to monitor individual molecular and biological processes [17]. These achievements have dramatically augmented our understanding of molecular oncology and this body of knowledge can now be translated into new drugs or agents for molecular imaging by allowing detection of patients with specific molecular profiles and improving patient care [20].

Finally a significant advance has been achieved with the theranostics which represents a research field integrating two distinct approaches that both encompass all steps of patients' management [21–23]. Of course, medical imaging is the prerequisite for such approach. However, the other mainstay of this approach is the use of molecular biomarkers which are important in the diagnostic processes, in determining the best course of treatment, in monitoring the patient's response and in detecting potential recurrence of the disease, and in anticipating potential adverse effects. Basically, theranostics has three distinct fields of application. They include (1) selection of patients for a specific treatment, (2) the prediction for drug response, resistance, and safety, and (3) monitoring of the therapeutic response [21–23].

This and much more are the heart of this special issue on the advances in diagnosis and treatment of prostate cancer. This special issue encompasses articles on the state of the art, advantages, and disadvantages, current limitations, and future perspectives of Pca monitoring and treatment methods. G. L. Gravina et al., *“Strategies for imaging androgen receptor signaling pathwayin prostate cancer: implications for hormonal manipulationand radiation treatment,”* D. Junker et al.,* “Evaluation of the PI-RADS scoring system for classifying mpMRI findings in men with suspicion of prostate cancer,”* S. F. Carbone et al.,* “Diffusion-weighted magnetic resonance diagnosis of local recurrences of prostate cancer after radical prostatectomy: preliminary evaluation on twenty-seven cases,”* and V. Panebianco et al.,* “Advanced imaging for the early diagnosis of local recurrence prostate cancer after radical prostatectomy,”* present advanced clinical and molecular imaging methods in clinical follow-up of response to therapy. T. Gondek et al.,* “Evaluation of 12-lipoxygenase (12-LOX) and plasminogen activator inhibitor 1 (PAI-1) as prognostic markers in prostate cancer,”* M. Srivastava et al.,* “Diverse effects of ANXA7 and p53 on LNCaP prostate cancer cells are associated with regulation of SGK1 transcription and phosphorylation of the SGK1 target FOXO3A,”* F. Zazzeroni et al., “*KCTD11 tumor suppressor gene expression is reduced in prostate adenocarcinoma,”* D. Gianfrilli et al.,* “Sex steroid metabolism in benign and malignant intact prostate biopsies: individual profiling of prostate intracrinology,”* M. Lanciotti et al.,* “The role of M1 and M2 macrophages in prostate cancer in relation to extracapsular tumor extension and biochemical recurrence after radical prostatectomy,”* I. Giusti and V. Dolo, “*Extracellular vesicles in prostate cancer: new future clinical strategies?,”* T. Van den Broeck et al.,* “The role of single nucleotide polymorphisms in predicting prostate cancer risk and therapeutic decision making,”* A. Dimakakos et al.,* “Novel tools for prostate cancer prognosis, diagnosis, and follow-up,”* and A. Irelli et al.,* “Bioclinical parameters driving decision-making of subsequent lines of treatment in metastatic castration-resistant prostate cancer,”* offer new insight into the use of some traditional and less well established cancer biomarkers in clinical and laboratory practice. The works of C. Festuccia et al.,* “Antitumor effects of saffron-derived carotenoids in prostate cancer cell models,”* S. Taurin et al.,* “A novel role for raloxifene nanomicelles in management of castrate resistant prostate cancer,”* and A. Colciago et al.,* “In vitro chronic administration of ERbeta selective ligands and prostate cancer cell growth: hypotheses on the selective role of 3beta-adiol in AR-positive RV1 cells,”* deal with the use of innovative pharmacological treatments. Of special interest are the articles that report on novel focal treatments, hypofractionated or modulated and intensified adjuvant radiation treatments for the management of prostate cancer, by A. M. Hirst et al., “*Low temperature plasma: a novel focal therapy for localized prostate cancer?,*” M. Valeriani et al.,* “Image-guided hypofractionated radiotherapy in low-risk prostate cancer patients,”* G. Mantini et al.,* “Intensified adjuvant treatment of prostate carcinoma: feasibility analysis of a phase I/II trial,”* S. Barra et al.,* “Image guided hypofractionated radiotherapy by helical tomotherapy for prostate carcinoma: toxicity and impact on Nadir PSA,”* and M. Mangoni et al.,* “Hypofractionation in prostate cancer: radiobiological basis and clinical appliance.”*
